# Supplementary figures and images for: The EAAT1 aspartate/glutamate transporter is dispensable for acute myeloid leukemia cell growth and response to therapy
Source: PLoS One. 2026 Feb 24;21(2):e0329048. doi: 10.1371/journal.pone.0329048 (PMC12931770; doi:10.1371/journal.pone.0329048)

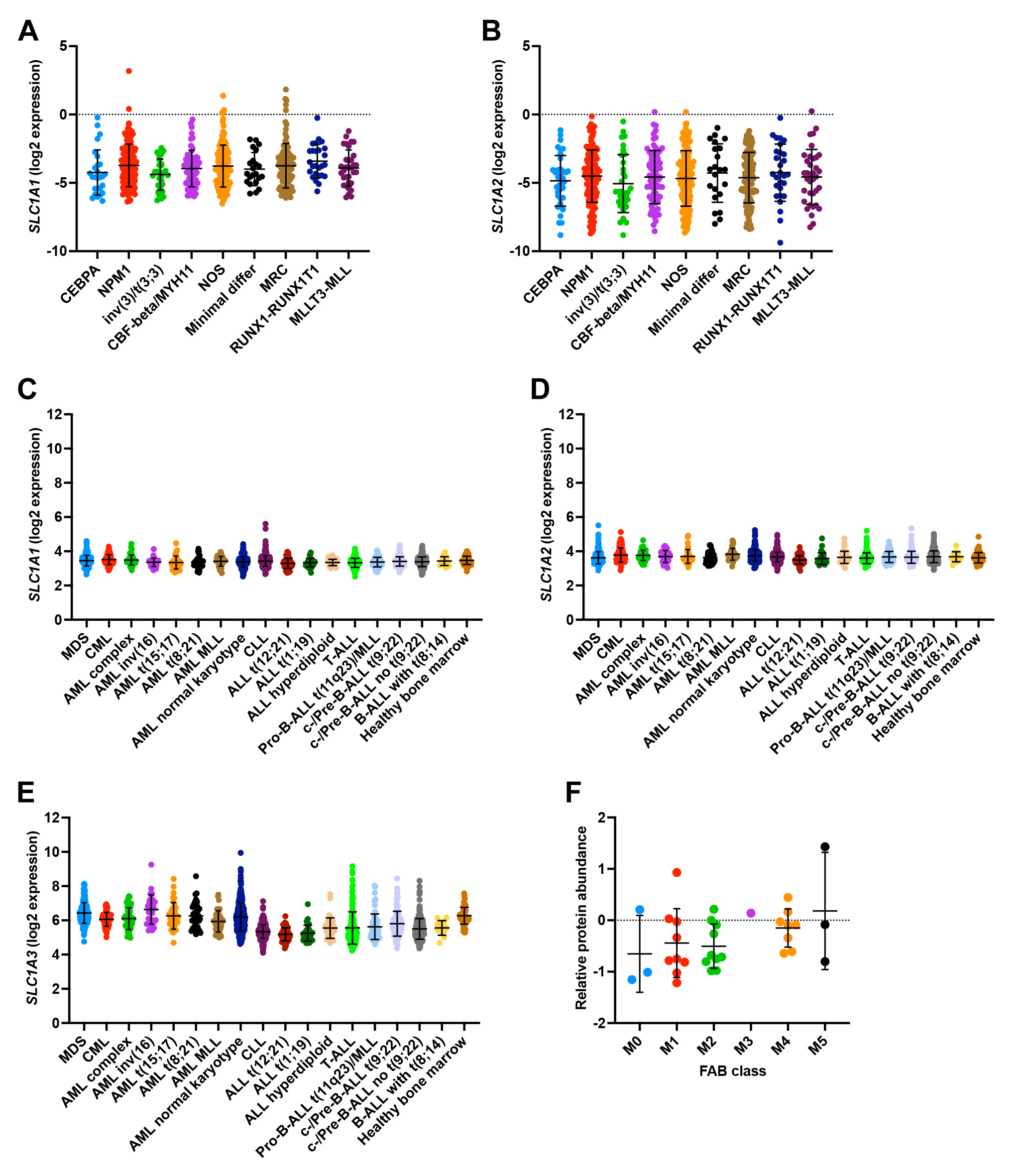

Supplement: S1 Fig — A-B. Expression of SLC1A1 (A) and SLC1A2 (B) in AML cells across different patient genetic subgroups in the BEAT-AML cohort. C-E. Expression of SLC1A1 (C), SLC1A2 (D) and SLC1A3 (E) in bone marrow cells of patients with different hematological cancers or healthy donors from the MILE study cohort. F. EAAT1 protein levels in bone marrow cells of AML patients stratified according to FAB classification in the TCGA cohort. (TIF) [file pone.0329048.s001.tif]

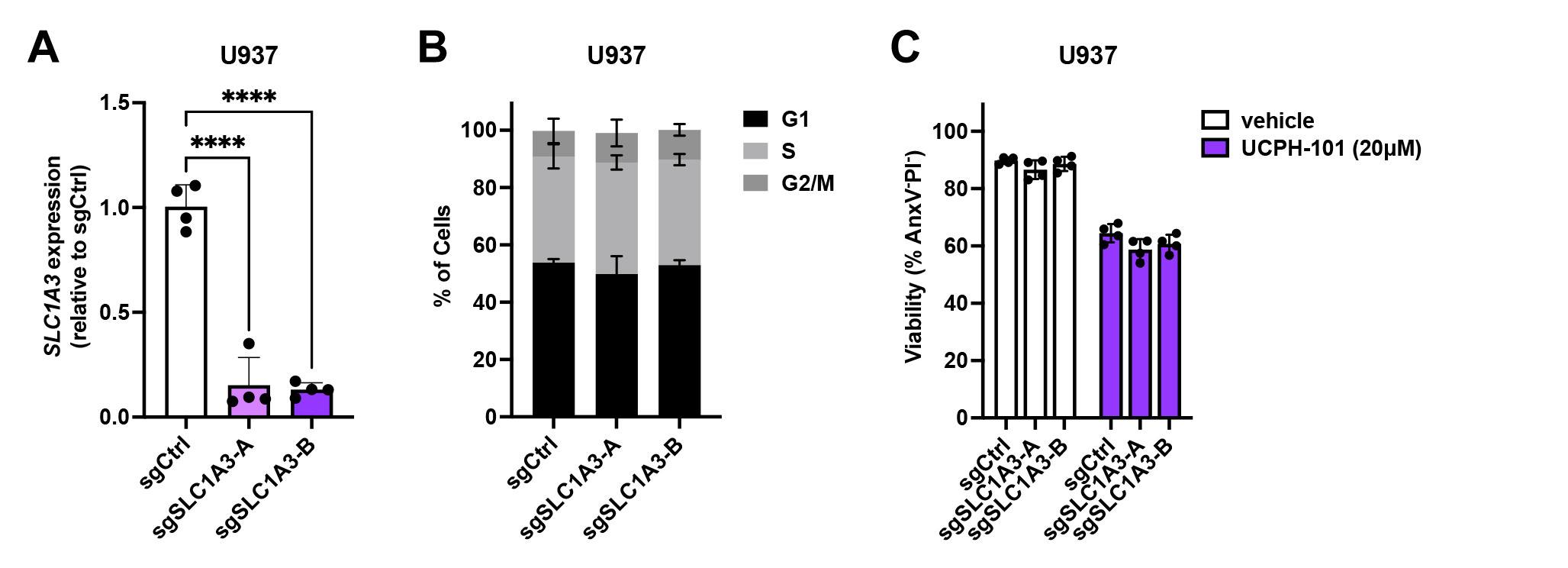

Supplement: S2 Fig — A. Expression of SLC1A3 in U937 cells after nucleofection with CAS9 protein and sgRNAs targeting SLC1A3 or a control sgRNA. Every dot represents a different clone measured in biological duplicate. B. Cell cycle analysis of U937 cells with or without SLC1A3 knockout as measured by flow cytometry. C. Viability of U937 cells with or without SLC1A3 knockout in the absence or presence of UCPH-101 (20 µM) for 24 hours, as measured by flow cytometry. Every dot represents a different clone measured in biological triplicate. Data are presented as mean ± SD. ****P < 0.0001. (TIF) [file pone.0329048.s002.tif]
